# Multimodal Assessment of Smoking cue Reactivity During a Smoking Cue Exposure Task

**DOI:** 10.1177/15500594221138273

**Published:** 2022-11-25

**Authors:** A.M. Kroczek, B. Schröder, D. Rosenbaum, A. Mühleck, J. Diemer, A. Mühlberger, A. J. Fallgatter, A. Batra, A.-C. Ehlis

**Affiliations:** 1 Department of Psychiatry and Psychotherapy, University Hospital of Tuebingen, Tuebingen, Germany; 2 Department for Clinical Psychology and Psychotherapy, Institute of Psychology, 9147 University of Regensburg, Regensburg, Germany; 3 kbo-Inn-Salzach-Hospital, Wasserburg am Inn, Germany

**Keywords:** substance use disorders, tobacco, cue reactivity, cue exposure, HRV, craving, beta power

## Abstract

*Background.* Cue-reactivity as a characteristic symptom of substance use disorders (SUD) is highly context dependent. Paradigms with high context validity need to be established for the investigation of underlying neurobiological mechanisms. While craving can be assessed by self-report as one aspect of cue-reactivity (CR), the assessment of biological measures such as the autonomous response and EEG promises a holistic perspective including CR at an automatized level. In a multimodal approach, smoking cue exposure (CE) effects on heart rate variability (HRV), EEG frequency power, and craving as well as their interrelation were assessed. This pilot study focused on the validity of CR measurements in a naturalistic CE paradigm. *Methods.* EEG frequency power, HRV, and craving were assessed during resting state (RS) and smoking CE in smokers (n = 14) and nonsmoking controls (n = 10) to investigate the psychophysiological and subjective reactions to CE. *Results.* Increased beta power was found only in smokers during CE compared to the control condition. There was an inverse correlation of beta power and maximum craving. Likewise, HRV correlated negatively with maximum smoking urges in smokers immediately after the measurements, without differentiation between CE and control condition. *Conclusion.* The increased beta power in smokers during CE is discussed as increased inhibitory control related to reduced craving in smokers. Furthermore, increased craving during CE seems to be associated to decreased vagal activity. The multimodal measurements during the CE showed ecological validity to be fundamental for CE assessment in clinical populations to evaluate its predictive value.

## Introduction

Substance use disorders (SUD) are characterized by high relapse rates; e.g., 12-month prevalence of smoking abstinence after a smoking cessation program is 25–40%,^1,2^ which is still unsatisfyingly low. Therefore, a better understanding of the underlying neurobiological mechanisms in SUD is necessary to provide new therapeutic approaches for relapse prevention. Prominent etiological models posit that SUD are characterized by an increasing imbalance between deliberate decision making and automatized behavior.^3^ Complementary to subjective craving, neurobiological correlates of SUD have been investigated^4^ and their predictive value for relapse has been evaluated.^5^

Neurophysiological correlates of SUD can be investigated as traits independent of a drug-related context (e.g. resting state) or as correlates of drug-related processing, e.g. in consumption-related tasks such as cue-exposure (CE). A review of frequency band power findings during resting state found no consistent results in SUD.^6^ However, there are studies reporting increased resting-state theta band activity in alcohol-dependent patients^7,8^ – discussed as neurobiological correlate of incentive salience during withdrawal and craving states – as well as increased beta power, which was interpreted as an imbalance of excitation-inhibition cortical homeostasis.^9^ However, to our knowledge, there is no study comparing both resting state and a consumption-related task. Therefore, the aim of our study was to fill this gap by investigating both resting-state brain activity and a consumption-related task (i.e., smoking cue exposure) in smokers compared to controls.

Smoking cue exposure activates the addiction memory, which can be measured as an automatized response termed ‘cue-reactivity’ (CR), which is closely related to relapse in SUD. CR evolves with conditioning processes induced by repeated consumption throughout the development of a SUD and is reflected on different levels: craving as the subjective level, reduced vagal and increased sympathetic activity at the autonomous level and finally approach behavior at the behavioral level.^10^ Although different CR levels are not fully intercorrelated, cue-induced craving relates to behavioral measures like an increased number of smoking puffs, puff volume and a shorter latency to smoke in smokers within the experimental setting.^11^

CR is very pronounced in smokers, as various everyday situations reflect conditioned cues, e.g., proximal consumption-related cues (e.g., a cigarette) or distal context cues (e.g., feeling stressed). Therefore, learning to apply coping strategies for CR is a psychotherapeutic intervention termed cue-exposure (CE) therapy.

From a neurobiological perspective on CE, CR is inhibited by brain regions involved in cognitive control; thus, relapse is related to decreases in cognitive control concurrent to CR increases.^12^ During CE, response inhibition for a prepotent response is required, e.g., lighting a cigarette. Theta power was related to premotor cognitive processes such as response program updating, while beta power was discussed as correlate of inhibitory control.^13^ During CE, increases in beta power in smokers were discussed in terms of an increased cortical arousal or processing bias for drug-related cues.^14,15^ Especially frontal EEG-activity has been found to differentiate smokers from non-smokers during smoking CE.^14,16^ Therefore, we will focus on EEG frequency band power at frontal electrodes to investigate differences between smokers and nonsmoking controls. Additionally, we will compare this frontal activity during CE to the frontal activity during resting state. This approach allows a direct inference on task specificity.

In our multimodal approach, measures of SUD-related brain activity were supplemented by heart rate variability (HRV) at the autonomous level. While high-frequency (HF) band power is related to vagal activity, low-frequency (LF) and the LF/HF ratio are related to sympathetic activity.^17^ HRV results in SUD populations indicate increased sympathetic^18^ and decreased parasympathetic activity during CE.^19^ Therefore, we expect higher LF band power and decreased HF band power in smokers during smoking CE.

Another focus of our pilot study is to establish a CR paradigm with high ecological validity. More realistic cue exposure approaches adopted to the clinical application of CE in SUD^20^ increase clinical relevance and ecological validity of CR results gained from the most often used passive picture viewing task.^20^ In order to improve this therapeutic approach, it is important to understand the neurobiological background during an active CE task. To our knowledge, no multimodal approach is reported for a direct comparison of a naturalistic smoking CE to controls and a neutral control condition. For the application in clinical populations, CR EEG measures during such realistic tasks need to be validated.

As paradigms in highly naturalistic settings are associated with increased artifacts, e.g. movement, feasibility of data analysis has to be shown. Most reported CR results are conducted in a highly standardized setting and averaged across trials before interpretation. In our new approach, we increase the ecological validity at the price of trial averaging, requiring suitable artifact correction strategies. For this pilot study, we decided to use independent component analysis as a convenient artifact correction approach^22^ for EEG frequency band power results during smoking CE. Our multimodal approach allows for a validation across methods and is therefore the basis for our analysis.

We already applied the CE paradigm using functional near-infrared spectroscopy (fNIRS), yet without the adjuvant control condition we included in the current study design.^23^ We showed that smoking CE induced craving and reduced parasympathetic activity (high-frequency heart rate variability [HF-HRV]), while fNIRS activity patterns reflected higher connectivity in prefrontal regions (OFC–dlPFC) in smokers. To further validate this approach with another method, we focused on EEG frequency bands in the current study. Although EEG has a lower spatial resolution than fNIRS, EEG reflects synchronized activity of neurons deeper than just the outer cortical layers, theta band has been localized to the cingulate cortex,^24^ e.g. which cannot be reliably measured with fNIRS. Tasks involving the handling with smoking cues were found to increase theta band power in smokers^24^; therefore, we expected theta to be increased during our smoking CE as well. In addition to Knott *et al*'s design, we conducted not only an active paradigm but also a resting-state measurement to control for non-task-specific effects in smokers.

Taken together, our first hypothesis is to show the validity of single-trial measures during smoking CE through the detection of differences in activity patterns of smokers and nonsmokers. As smoking inhibition requires cognitive control, we expected increased beta band power in the time course of CE, especially for smokers and especially for the smoking-related (but not the neutral control) condition. Second, we aim at a clarification of the inconsistent resting-state data; to this end, we included a baseline resting- state measurement before smoking CE. Another resting-state period after the experiment promises some information on effects of smoking CE on resting-state measurements. Finally, we focused on correlations of multimodal measures in smokers to capture a multilevel CR.

## Methods

### Participants

Overall, 29 participants conducted the neurophysiological measurements; data from 3 participants needed to be discarded due to technical problems, and data from 2 participants because of the heavy artifact load, resulting in a final sample of 14 smokers (9 female) and 10 nonsmokers (6 female) without current mental, neurological, or chronic internal diseases according to self-disclosure. Smokers (aged 18-45 years, *M* = 27.6, *SD* = 7.8) were recruited by mailing list at the University and University Hospital Tuebingen and confirmed smoking on a regular basis (daily smoked cigarettes: range 6–25, *M* = 17, *SD* = 10 cigarettes/day). Nonsmoking controls (age: *M* = 23.5, *SD* = 4.3) reported having smoked a maximum of 5 cigarettes during their lifetime. Smokers started smoking between the age of 11 and 19 (*M* = 16, *SD* = 3). Fagerström Test for Nicotine Dependence^25^ scores (FTND) ranged from 0–6 in smokers (*M* = 3*, SD* = 2). FTND scores reveal smokers without nicotine dependence, reflected in FTND scores 0–2. Smokers reported their last cigarette in a range between 15 min and 38 h (*M* = 7 h, *SD* = 14), revealing a large variance considering nicotine withdrawal, as participants were not instructed to limit or control their smoking behavior before the measurement. The study was approved by the Ethics Committee of the Faculty of Medicine and written informed consent was given by all participants in accordance with the Declaration of Helsinki in its latest version.

### Procedure

**Figure 1.**
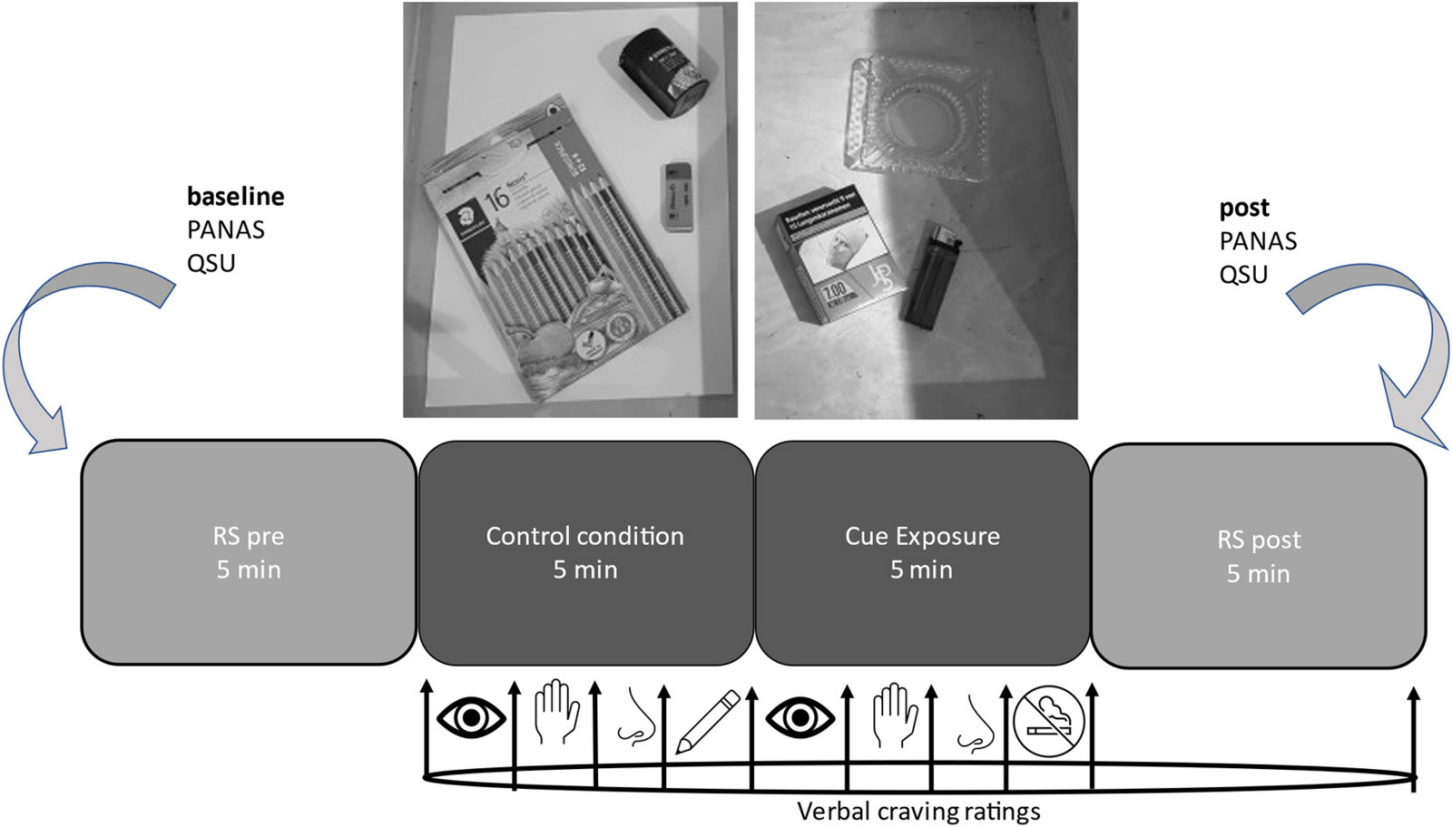
Overview of the experimental design. Positive and Negative Affect Schedule (PANAS) and Questionnaire on Smoking Urges (QSU) were assessed as baseline measure and after the experiment. A 5 min resting-state (RS) sequence was the first and last measurement. During the 5 min control condition, box A (including pens, sharpener, rubber, white paper) was inspected visually, tactilely, and olfactorily, ending with common movements without writing on the paper. Equivalently, in the cue exposure condition, box B (including cigarettes, a lighter, an ashtray) was inspected visually, tactilely, and olfactorily, ending with common movements without smoking the cigarette. Ten verbal craving ratings (0–100) were assessed at the marked points.

During the application of EEG and electrocardiogram (ECG), participants filled out paper-pencil questionnaires assessing sociodemographic and smoking-related data. Immediately before the experiment and directly thereafter, the Positive and Negative Affect Schedule PANAS,^26^ and a smoking-related craving questionnaire QSU^27^ were conducted to capture state effects affected by CR. Then the first task was instructed, a seven minute approach-avoidance task, where smoking-related and control pictures had to be pushed away or pulled towards the own body using a joystick (depending on the content of the pictures, i.e., smoking-related or neutral). This task consisted of 6 blocks of 30 s duration each; blocks differed regarding the task instruction (i.e., approach or avoid smoking-related cues) and were presented in randomized order. Data will be analyzed in an event-related fashion for the four resulting conditions (approach/avoid smoking/non-smoking-related cues) and reported elsewhere (Schroeder *et al*, in preparation) since this more conventional study design and analysis does not fit the current topic (of implementing a naturalistic CE condition) well. Thereafter, craving was rated by every participant on a 0 to 100 scale (0 no craving at all, 100 most intense craving ever experienced), with a total of 10 values within the following 20 min of the resting-state (RS) and CE task. For the following 5 min, participants were instructed to just follow their thoughts during the eyes-open RS measurement. In the following, two closed boxes labeled A (control condition) and B (experimental condition) were positioned on a table in front of the participants. In the following 5-min control condition the neutral item exposure task began with the opening of box A, including instructions for visual, tactile, and olfactory inspection of the neutral items (pens, white paper, rubber, sharpener). The task ended after the instruction to use the items as usual without drawing something on the paper. Thereafter, the equivalent 5 min smoking CE followed with the opening of box B. During CE, participants were instructed for visual, tactile and olfactory inspection with smoking-relevant cues, reflecting an intensified confrontation ending with typical movements, except for lighting and actually smoking the cigarette. Finally, another 5-min RS period with open eyes was measured. During control and experimental tasks, craving was assessed after each increase of task intensity, resulting in 10 verbal craving ratings within 20 min of total duration of the experiment; see Figure 1 for an overview (only smokers are depicted as controls rated craving levels with 0 throughout). This CE task was already applied in an fNIRS study.^23,28^

### EEG

EEG was recorded with a stationary BrainAmp DC amplifier (Brain Products GmbH, Gilching, Germany) and the BrainVision Recorder Software (Brain Products GmbH, Gilching, Germany) at a 1000 Hz sampling rate with 19 passive EEG electrodes placed according to the 10–20 standard sites (Fp1, Fp2, F7, F3, Fz, F4, F8, T7, C3, Cz, C4, T8, P7, P3, Pz, P4, P8, O1, O2) and three additional channels for eye movement correction.^29^ An online notch filter (50 Hz) was applied and impedances were kept below 10 kΩ throughout the measurement. Data was preprocessed (BrainVision Analyzer, Version 2.2.0, Brain Products GmbH, Gilching, Germany), involving downsampling to 500 Hz and bandpass filtering (0.5–40 Hz), with the increased high-pass filter to prevent negative effects on the independent component analysis (ICA) evaluation. Data was segmented into four conditions (RS baseline, control condition, experimental condition, RS post).

Further analysis was conducted in EEGLAB (Delorme and Makeig, 2004), a Matlab (The MathWorks Inc., Natick, USA) toolbox. Using manual artifact rejection, epochs and channels with strong artifact load were rejected from the data set. ICA was conducted and thereafter components were automatically labeled and rejected if probability of a component for brain contribution was below 5%. For the ICA analysis, a whole brain EEG was required, although only frontal electrodes (F3, F4, Fz) were subsequently extracted for power spectrum density analysis. After ICA, data was re-referenced offline to a common average, reusing the online reference as FCz in the latter. Each condition was cut into 1 s epochs to conduct power spectrum density analysis, extracting the following frequency bands at F3, Fz and F4: delta (0.5–4 Hz); theta (4–8 Hz); alpha (8–12 Hz); beta (13–30 Hz). Due to the high movement artifact burden, a low high-frequency cut-off was chosen for bandpass filtering (40 Hz); therefore, gamma band (30.5–60 Hz) was excluded from the analysis. Due to the skewness, data was log-transformed.

### HRV

Electrocardiogram was acquired using BrainAmp ExG amplifier and BrainVision Recorder software (Brain Products GmbH, Gilching, Germany) at 500 Hz, using two AG/AgCl electrodes applied to the right shoulder and left chest. Kubios 2.0 (Biosignal Analysis and Medical Imaging Group, University of Finland) was used with (?) semi-automatic medium artifact detection and R peak detection extracting low and high frequency band power peaks of HRV. Analyzed parameters were heart rate (HR), low (LF) and high frequency (HF) power.

### Statistical Analysis

Repeated measurement ANOVAs (RM-ANOVA) were calculated for frequency band power and HRV separately with task (rest vs active) and experiment-half (first neutral vs second CE half) as within-subject factors and group (smokers vs controls) as between-subject factor (using IBM SPSS Statistics 21). Only the highest-level interactions involving cue, response or group are reported; Greenhouse-Geisser correction was used whenever necessary. Normal distribution of the data was tested by Kolmogorov-Smirnov tests. Non-parametric testing was used for variables deviating from normal distribution. Threshold for statistical significance was α = 0.05.

For statistical analysis of EEG frequency band power, we focused on the frontal electrodes (F3, Fz, F4) for further analysis in a 3 × 2 × 2 × 2 (electrode × task × experiment-half × group) ANOVA. There was a main effect of electrode in every frequency band. Considering the aim to increase the power of our small pilot sample, Fz – the electrode with the largest effect – was focused in the new (reduced) task (active vs rest) × timing (first half vs second half) × group (smokers vs controls) ANOVA model.

PANAS was analyzed in a 2 × 2 × 2 RM-ANOVA with timing (before vs after the experiment) × affect (positive vs negative) as within subject factors and group (smokers vs controls) as between-subject factor. QSU was analyzed for smokers using a t-test comparing values before and after the experiment. Maximum craving was derived from the ten craving ratings across the experiment. Spearman correlation was applied to correlate significant frequency band group effects to subjective level (craving) and autonomous response (HRV).

## Results

### Behavioral Measures

For PANAS scores, there was an interaction of time × affect × group, *F_1, 21_* = 4.578, *P* = .044, *η²* *=* *.*18. Positive affect decreased in smokers (pre: *M* = 26.3, *SD* = 6.9; post: *M* = 21.6, *SD* = 7.1) without significant changes in negative affect (pre: *M* *=* 13.1, *SD* = 3.4; post: *M* = 13.91, *SD* = 4.7). There was no significant effect in nonsmokers (*P* *=* *.*631).

**Figure 2.**
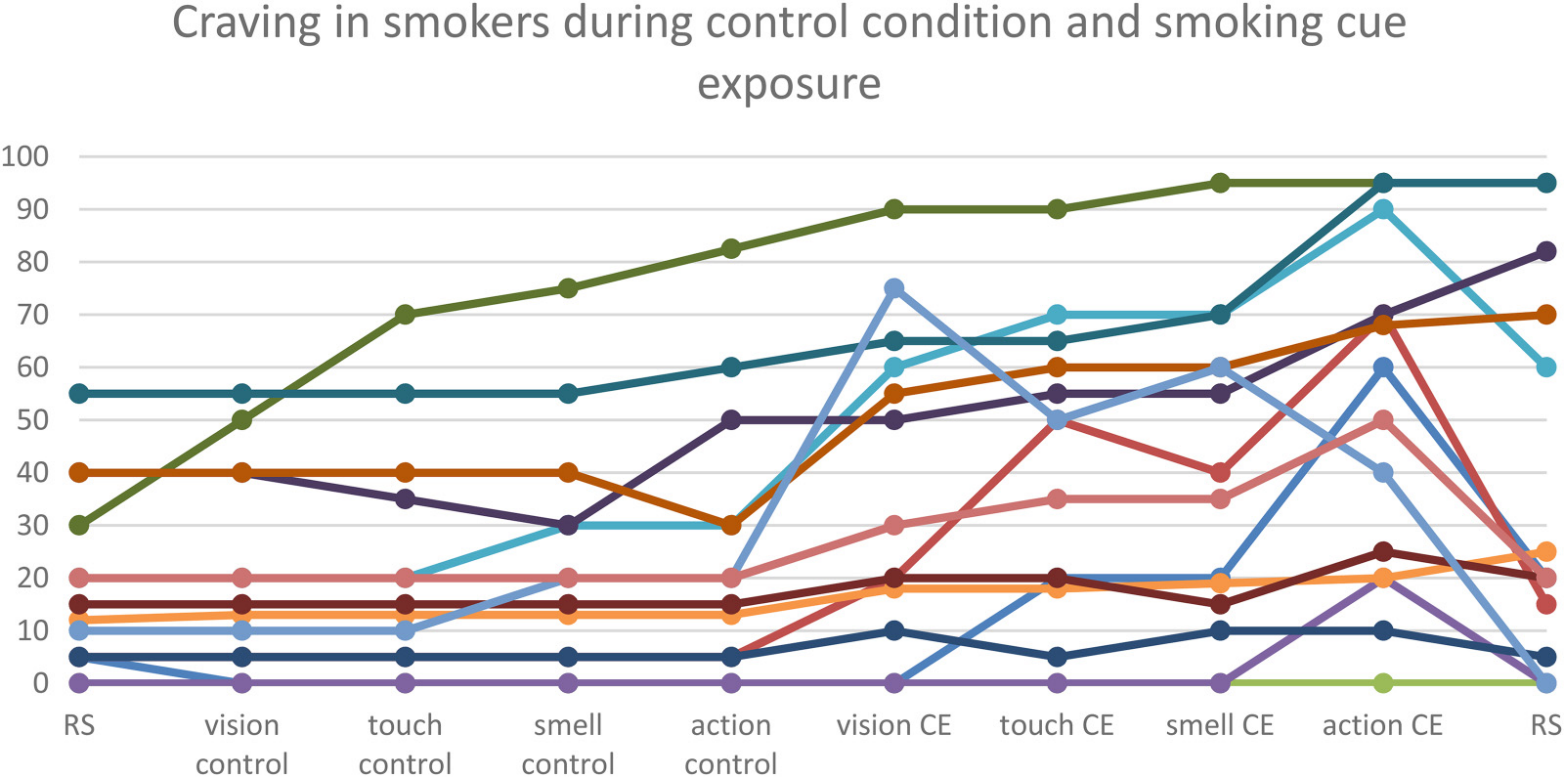
Individual craving ratings (0–100) for smokers (n = 14). Each individual is depicted as a separate line in the time course of the experiment. Smokers were asked to rate their urge to smoke on a scale from 0 (no craving at)–100 (maximum craving). In the beginning and at the end of the measurement there was a 5-min resting state measurement. Both the 5-min control condition and the 5-min cue exposure (CE) included “vision”, ”touch”, “smell” of and “interaction” with the items in the box. For most of the individuals, maximum craving was assessed when making smoking movements without lighting the cigarette.

Nonsmoking controls rated craving to smoke throughout the experiment with 0 (except for one participant who rated one item with 20). For smokers maximum craving (from the total of 10 ratings) ranged from 0 to 95; see Figure 2 for the individual time courses (*F_1, 61_* = 7.696, *P* < .001, *η²* *=* *.*235).

Smoking urges according to QSU, which was only assessed in smokers, increased from baseline (*M* *=* 20.6, *SD* = 10.6) to the end of the experiment (M = 29.1, SD = 12.2), *t_13_* = 2.53, *P* = .025, *d* *=* *.*427.

### Power Frequency Analysis

There was a task effect on delta power at Fz (*F*_1, 11_ = 5.550, *P* = .028, *η²* *=* *.201*) revealing increased power during CE (*M* = 0.12, *SD* = 2.27) compared to RS (*M* = 0.05, *SD* = 2.56) in the whole sample. For theta, there was a task effect (*F_1, 22_* = 5.703, *P* = .026, *η²* *=* *.*21) indicating higher activity in RS (*M* *=* -0.37 *SD* = .25) compared to CE (*M* = -.44, *SD* = .24). Regarding alpha power, there was a main effect of both task (F_1, 22_ = 10.29, *P* = .004, *η²* *=* *.32*) and experiment-half (F_1, 22_ = 9.05, *P* = .006, *η²* *=* *.29*). Alpha decreased from RS (*M* = -0.34, *SD* = 0.43) to CE (*M* = -0.51, *SD* = 0.29) and increased from the first half (*M* = -0.46, *SD* = .35) to the second half (*M* *=* *-0.*39, *SD* = 0.35).

**Figure 3.**
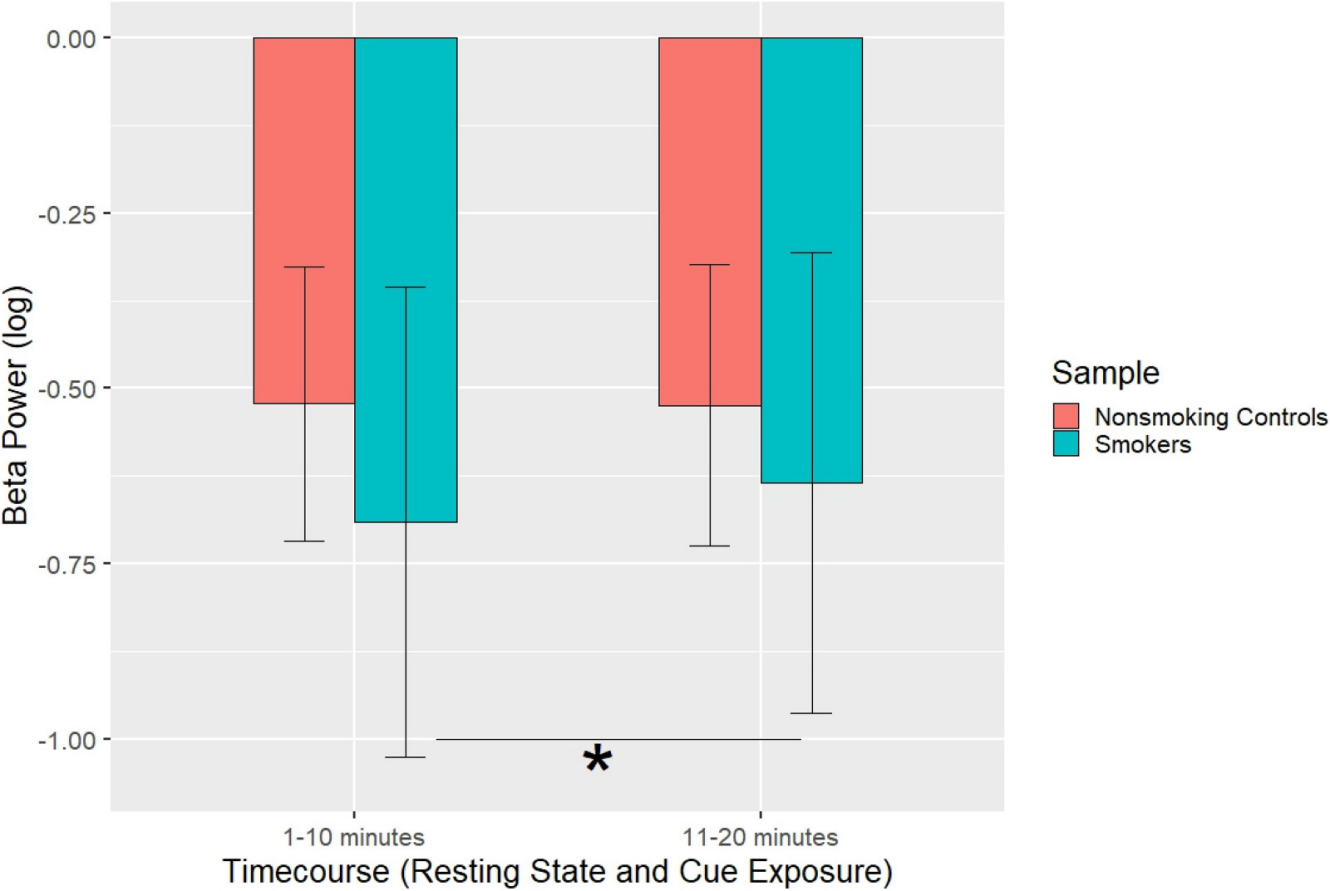
The log-transformed beta power (y-axis) is shown for both groups (controls and smokers) comparing the first and second half of the experiment for each group (x-axis). The first half of the experiment subsumes RS pre and the control condition while second half consists of the experimental condition and RS post. Beta power increased significantly in smokers from the first half to the second half of the experiment. There was no significant difference in controls.

For beta power, there was a main effect of experiment-half (F_1, 22_ = 5.364, *P* *=* *.*039, *η²* *=* *.20*) and task (*F_1, 22_* = 6.03, *P* = .022, *η²* *=* *.22*). Furthermore, there was a group × experiment-half interaction (*F_1, 22_* = 6.40, *P* = .019, *η²* *=* *.23*). While beta power did not differ between first (*M* *=* *-0.*522, *SD* *=* *0.*18) and second (*M* = -0.52, *SD* *=* *0.*19) half of the experiment in nonsmokers (*P* = .583), there was a significant effect for smokers (*t_13_* = 3.27, *P* = .006, Cohen's *d* = .849), with an increase in beta power from the first half (*M* *=* *-.*690, *SD* *=* .325) to the second half (*M* *=* *-.*634*, SD* *=* *.*315), see Figure 3.

### HRV

Regarding HF peak as measure, there was a main effect of task (*F_1, 22_* = 8.18, *P* *=* *.009, η²* *=* *.27*). HF peak was increased during RS (*M* = 0.24, *SD* = 0.05) compared to CE (*M* = 0.21, *SD* = 0.05). Furthermore, there was an experimental-half × task × group interaction, *F_1, 22_* = 5.53, *P* *=* *.028 η²* *=* *.20.*

**Figure 4.**
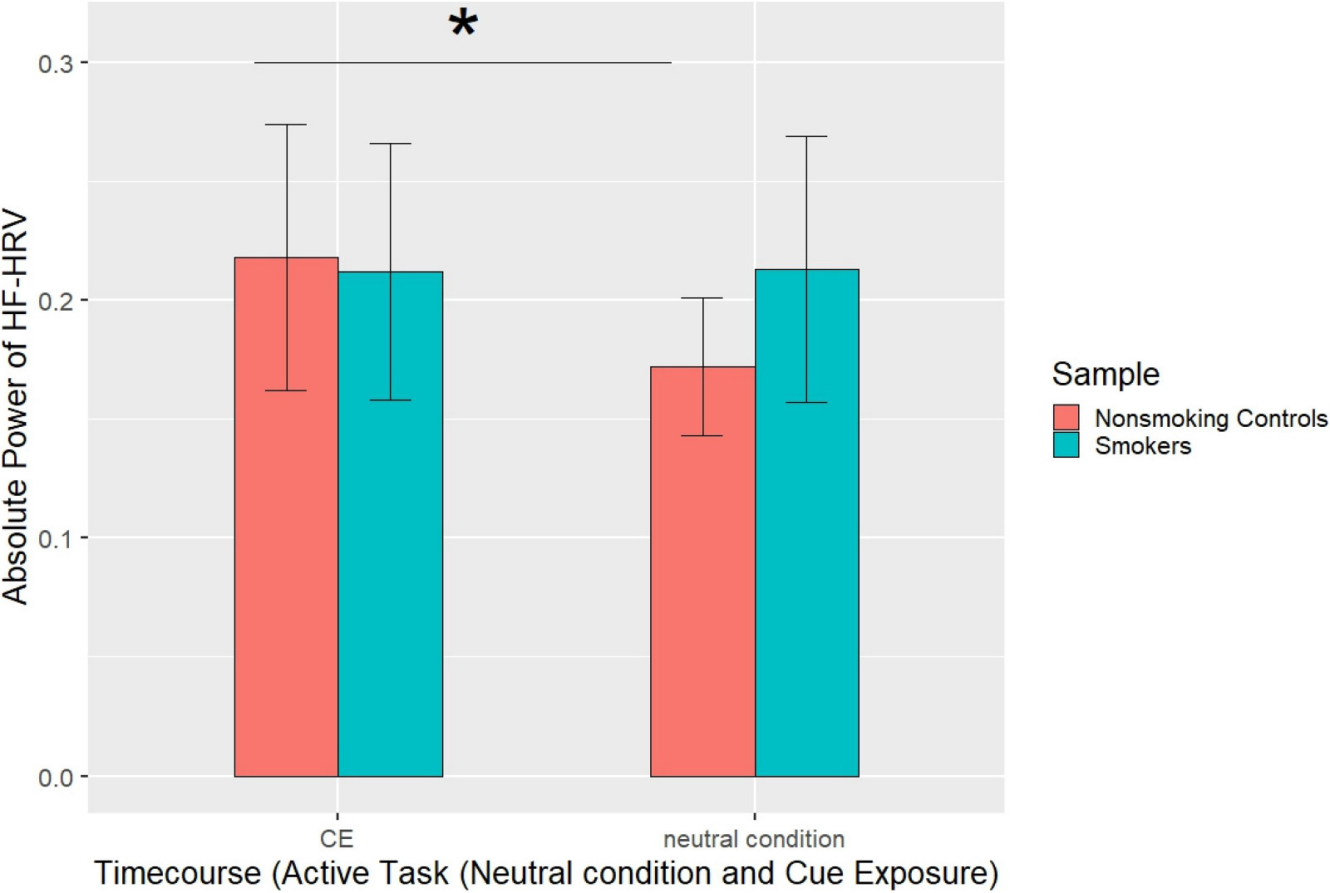
HF HRV reflecting vagal activity in controls and smokers during control condition and smoking ce. A significant difference was revealed in controls with lower HF during the control condition as compared to CE. There was no difference in smokers.

Post-hoc analysis revealed an experimental-half × group interaction for the active task *F_1, 22_* = 5.78, *P* *=* *.025, η²* *=* *.21*, but not for resting state *(P* *=* *.123).* In controls (*t_9_* = 2.34, *P* = .044, *d* = .74), the first half of the experiment (control condition: *M* *=* 0.17, *SD* = 0.03) differed from the second half (CE: *M* = 0.22, *SD* = 0.06), whereas smokers revealed no difference between control (*M* = 0.21, *SD* = 0.07) and experimental (*M* = 0.21, *SD* = 0.05) condition*, t_13_* = 0.13, *P* = .897, *d* = .035, see Figure 4.

### Correlations

In smokers, maximum craving correlated negatively with HRV-HF peak during the control and experimental condition (see Table 1). Likewise, beta power and maximum craving correlated negatively. There was no correlation of beta band power and HF-HRV in either smokers (all *P* *>* *.*117) or controls (all *P* > .177). QSU before the measurement correlated negatively with HRV during CE, while RS HF-peak correlated with QSU rating after the measurement.

**Table 1.** Correlations of Craving Ratings and Neurobiological Markers in Smokers.

	Beta first RS + control	Beta second CE + RS	HRV first RS	HRV Control	HRV CE	HRV second RS
Maximum craving	*r* = -.539* P = .047	*r* = -.586* P = .028	*r* = -.391 *P* = .167	*r* = -.613* P = .020	*r* = -.561* P = .037	*r* = .181 *P* = .537
QSU pre	*r* = -.418 *P* = .137	*r* = -.406 *P* = .149	*r* = -.536* P = .036	*r* = -.333 *P* = .244	*r* = -.610* P = .020	*r* = -.036 *P* = .902
QSU post	*r* = -.248 *P* = .393	*r* = -.245 *P* = .399	*r* = .035 *P* = .906	*r* = .010 *P* = .973	*r* = -.100 *P* = .733	*r* = -.555* P = .040

*p < .005.

## Discussion

The aim of our pilot study was to establish a paradigm and to identify ecologically valid parameters for cue reactivity (CR) at different levels in a multimodal design. Our smoking CE paradigm elicited CR in smokers, as measured by craving ratings. Furthermore, smoking CE increased beta band power in smokers during CE and the consecutive resting-state measurement. We did not find any differences between smokers and controls in the remaining frequency bands. Theta and alpha band power were increased during resting state, while delta was increased during CE. For HRV, overall HF peak was increased during resting state compared to the active task. Nevertheless, we found an unexpected effect for controls, revealing more parasympathetic activity during smoking CE than in the control condition. Higher craving ratings correlated with lower parasympathetic activity as well as lower beta band power in smokers. The assessment of smoking urges (QSU) before CE correlated with craving ratings during the smoking CE, while high QSU ratings after the measurement correlated with lower vagal activity during resting state after CE.

One interpretation of increased beta power is increased inhibitory control during CE, which has been reported in other cognitive domains such as thought suppression^30^ and memory suppression of a motor task.^31^ Alcohol-dependent patients showing an increased event-related potential reflecting inhibitory processes (P3b) throughout an alcohol detoxification program exhibited higher abstinence rates in a 3-month follow-up.^32^ Interestingly, although measures of inhibitory control differ between smokers and nonsmoking controls, it is likely that this represents a functional mechanism compensating for CR effects. As our sample is very small, correlations have to be interpreted with caution, yet the inverse correlation of craving and inhibitory control fits well with the current literature on inhibitory control of CR, indicating that maximum craving is regulated by inhibitory control, or vice versa, less inhibitory control is necessary to inhibit weaker CR.

Our smoking sample had a great variance according to their smoking pattern; however, due to the small sample size, unfortunately, there was no possibility to investigate the effect of individual smoking patterns on the interrelation of the assessed CR measures. Nevertheless, it is highly interesting for future research to investigate the predictive value of those measures for relapse in a longitudinal approach. Investigations in this field could provide insights into the underlying mechanisms for effective treatment and therefore allow its directed modulation.

The prerequisite for such clinical studies is the identification of a valid paradigm. In our data, results indicated the need to consider the length of CR in relation to CE duration. Increased beta power lasted throughout the resting state after CE in smokers. This finding is sound from the therapeutic perspective of CE therapy, indicating the relevance of time for the decline of CR. In our previous studies, a 15-min CE was too short to fully capture the CR decline,^23^ which then was assessed within a 20-min CE epoch.^28^ Therefore, it is in line with our previous findings that inhibitory control of CR goes beyond the five minutes period in the current experiment, persisting even when the smoking cue is out of sight. Craving ratings after the measurement correlated with beta power during the second RS. Even without instruction, beta power was related to maximum craving in smokers after the CE.

In the current study, we advanced the CE paradigm implementing a control condition requiring the handling of neutral stimuli (writing tools) with similar size requiring motor action. However, as the order of stimulus presentation was fixed due to the expected prolonged CR duration, sequence effects cannot be excluded. To date, there is no convincing method to prevent carryover effects for a counterbalanced design,^33^ without taking the disadvantages of multi-session measurements. Nevertheless, control participants did not reveal significant beta power increases related to smoking CE as smokers did, so beta increase appears to be related to smoking habit.

For the correlations of smoking habits with the physiological markers, only the small smoker half of the sample was available. Nevertheless, increased beta power correlated with decreased maximum craving ratings, supporting the hypothesis of increased beta power related to decreased CR, possibly due to inhibitory processes. On the autonomous level of CR, we did not find differences between the control condition and CE in smokers. Interestingly, HF-peak related to vagal activity increased from the neutral task to CE in controls. Blunted HF-peaks were expected in smokers especially in a stressful situation^34^ such as CE, which was not shown by our own data. The validity of HF-peak as a measure of vagal activity was approved by increases during resting state compared to the active condition. Although there was no significant difference between the control condition and CE in smokers, vagal activity during CE and the control condition was negatively correlated with maximum craving, in accordance with the hypothesis that vagal activity is reduced during CE in smokers. This inverse correlation has been reported before in opioid craving.^35^ This highlights the importance of a dimensional approach for CE assessment to capture all consumption-related effects.

Overall, our results confirm the external validity of our EEG measures, despite the high movement-related artifact load during the paradigm. ICA artifact correction using a constant threshold for automatized component classification was a convenient method for our sample. Subjects that had to be excluded due to bad data quality were comparable to studies without such high risk of movement artifacts. This data reveals the practicability of EEG data analysis in studies with high ecological validity.

Alpha power was higher during RS compared to CE yet increased throughout the experiment in the whole sample. One hypothesis is increased tiredness of study participants over the course of the task. Unfortunately, there is no information what participants actually did during the RS epoch, e.g., if they thought about the experiment or managed to stay present detaching from upcoming thoughts. Asking such questions, authors found that self-referential thoughts during RS were related to increases in alpha activity.^36^ Our data revealed that theta activity was increased during RS compared to CE. Frontal theta was previously reported as a correlate of the default mode network.^37^ Increased theta power during RS therefore is in line with findings of default mode network activity. Unfortunately, we cannot provide qualitative data for the participants’ description of their RS experience.

Nevertheless, we had quantitative subjective mood data before and after the experiment. Positive affect decreased in smokers throughout the experiment, whereas there was no effect in controls. We cannot conclude from our data whether the CE per se is aversive, or the effect is closely related to withdrawal from nicotine, which is featured by negative mood. The pilot character with a small sample size is a limiting factor to investigate this question. Smoking habits in our sample were very diverse. Some smokers reported a severe smoking pattern, while others reported smoking on a non-daily basis. Due to the small sample size, we could not include time since the last smoked cigarette in our statistical model, which nevertheless is highly relevant for clinical populations and should be included in future studies. Furthermore, not every smoker in our sample had a nicotine dependency, depicted in FTND scores below 2. For future studies, a minimum FTND score should be considered to involve a more homogenous sample.

We used this multimodal approach to capture CE in smokers to validate the approach and its analytic strategy in a small pilot study in smokers compared to nonsmoking controls. The next step, however, is to extend this approach to its clinical application. Therefore, our results can contribute to a personalized medicine approach,^38^ a future perspective for multimodal assessments of CR. In larger populations of treatment-seeking individuals, this multimodal approach can be applied to investigate its prognostic value, e.g., when identifying subgroups prone to relapse and contributing to individualized medicine in terms of – for example – determining individuals profiting from CE.

Furthermore, larger studies will provide enough power to extend the analysis to include more channels. Furthermore, correlations in a larger sample will be more significant, and applied in a clinical sample analysis on the prognostic value of these measures will be possible.
